# Integrating brain, behavior, and phylogeny to understand the evolution of sensory systems in birds

**DOI:** 10.3389/fnins.2015.00281

**Published:** 2015-08-11

**Authors:** Douglas R. Wylie, Cristian Gutiérrez-Ibáñez, Andrew N. Iwaniuk

**Affiliations:** ^1^Neurosciences and Mental Health Institute, University of AlbertaEdmonton, AB, Canada; ^2^Lehrstuhl für Zoologie, Technische Universität MünchenFreising-Weihenstephan, Germany; ^3^Department of Neuroscience, University of LethbridgeLethbridge, AB, Canada

**Keywords:** principle of proper mass, wulst, lentiformis mesencephali, isthmo-optic nucleus, somatosensory specializations, prv, brain–behavior relationships, sound localization

## Abstract

The comparative anatomy of sensory systems has played a major role in developing theories and principles central to evolutionary neuroscience. This includes the central tenet of many comparative studies, the principle of proper mass, which states that the size of a neural structure reflects its processing capacity. The size of structures within the sensory system is not, however, the only salient variable in sensory evolution. Further, the evolution of the brain and behavior are intimately tied to phylogenetic history, requiring studies to integrate neuroanatomy with behavior and phylogeny to gain a more holistic view of brain evolution. Birds have proven to be a useful group for these studies because of widespread interest in their phylogenetic relationships and a wealth of information on the functional organization of most of their sensory pathways. In this review, we examine the principle of proper mass in relation differences in the sensory capabilities among birds. We discuss how neuroanatomy, behavior, and phylogeny can be integrated to understand the evolution of sensory systems in birds providing evidence from visual, auditory, and somatosensory systems. We also consider the concept of a “trade-off,” whereby one sensory system (or subpathway within a sensory system), may be expanded in size, at the expense of others, which are reduced in size.

## Introduction

Comparative anatomy of sensory systems has played a major role in developing theories and principles central to evolutionary neuroscience. As a simple example, lateral inhibition was first described in the ommatidia of the horseshoe crab (*Limula* sp.) (Hartline and Ratliff, 1972; Fahrenbach, 1985), but is essential to our understanding of visual processing in mammals and other vertebrates. Modern comparative neuroanatomy often uses multispecies data sets in which attempts are made to understand the evolution of specific behaviors and the correlated evolution of the brain and behavior. The latter studies, comparative studies of brain–behavior relationships, have flourished in recent years as a result of increased interest in understanding how the brain has evolved, (Striedter, 2005) as well as the development of advanced statistical methods to explore evolutionary patterns (Felsenstein, 1985; Harvey and Pagel, 1991; Garland et al., 1993; Pagel, 1999; Revell, 2010). These studies range in scope from analyses of relative brain size in relation to various life history variables and behaviors (e.g., Iwaniuk et al., 2001, 2004; Lefebvre et al., 2004; Pérez-Barbería et al., 2007; Sol et al., 2007, 2008) to the size of brain regions in relation to specific behaviors (Barton et al., 1995; e.g., Barton, 1998; Pellis and Iwaniuk, 2002; Sherry, 2006; Lindenfors et al., 2007). These kinds of studies have not been exempt of criticism. Healy and Rowe (2007) for example, suggested that correlations between behavioral or ecological factors and relative brain size are meaningless because the brain is composed of multiple, distinct functional units, and therefore changes in the size of the entire brain tell us little about the relationship between brain and behavior. At the same time, these same authors point out that, on the other hand, studies of specific sensory or motor regions, with clear defined function are much more useful as they can point out directly when and where selection is acting upon neural structures.

An inherent assumption of this type of correlational approach to brain–behavior relationships is that larger means better; i.e., that a bigger relative volume results in a better and faster processing of information. This principle is known as the “principle of proper mass” (Jerison, 1973), which states that the size of a neural structure is a reflection of the complexity of the behaviors that it subserves. While Jerison did not explicitly differentiate between absolute and relative size (Striedter, 2005), it is now widely accepted that more complex behavior means a larger relative size and not absolute size (but see Deaner et al., 2007 and Azevedo et al., 2009 for a discussions of the importance of absolute brain size in relation to cognition in mammals). Differences in relative volume of a neural structure are usually thought to reflect an increase in the number of neurons. Even though a positive correlation between volume and cell numbers has only been shown for particular neural structures a few times (Moore et al., 2011; Gutiérrez-Ibáñez et al., 2012), the total brain volume correlates well with the total number of neurons and appears to be one of the main factors that explains differences in relative brain size (Herculano-Houzel et al., 2007; Herculano-Houzel, 2009). Variation in neuronal numbers is not, however, the only factor explaining differences in the relative size of neural structures. For example, in some songbirds, seasonal changes in volume of song control brain nuclei involved in song learning are also associated with changes in neuron soma area (e.g., Tramontin et al., 2000; Thompson and Brenowitz, 2005) and dendritic structure (Hill and DeVoogd, 1991). Thus, differences in relative brain region size can arise from adding neurons or increasing the size of neurons.

Certainly the size of structures within the sensory system is not, however, the only salient variable in the evolution of sensory systems. The evolution of the brain and behavior are intimately tied to the evolutionary history of the species being examined (Harvey and Pagel, 1991; Striedter, 2005; Sherry, 2006). The vast majority of modern comparative studies therefore examine allometry, species differences in relative brain region size and brain–behavior relationships within a phylogenetic context, which enables a more accurate and holistic view of brain evolution (Iwaniuk, 2004; Striedter, 2005). Birds have proven to be a useful group for these studies because of widespread interest in their phylogenetic relationships (Hackett et al., 2008; Jarvis et al., 2014), the diversity of their sensory capabilities, and a wealth of information on the functional organization of most of their sensory pathways (Zeigler and Bischof, 1993; Dubbeldam, 1998; Dooling and Fay, 2000).

In this review, we examine the principle of proper mass in relation differences in the sensory capabilities among birds. We discuss how neuroanatomy, behavior, and phylogeny can be integrated to understand the evolution of sensory systems in birds providing evidence from visual, auditory and somatosensory systems. We also consider the concept of a “trade-off,” whereby one sensory system (or subpathway within a sensory system), may be expanded in size, at the expense of others, which are reduced in size.

## Visual systems in birds

**Figure 1:**
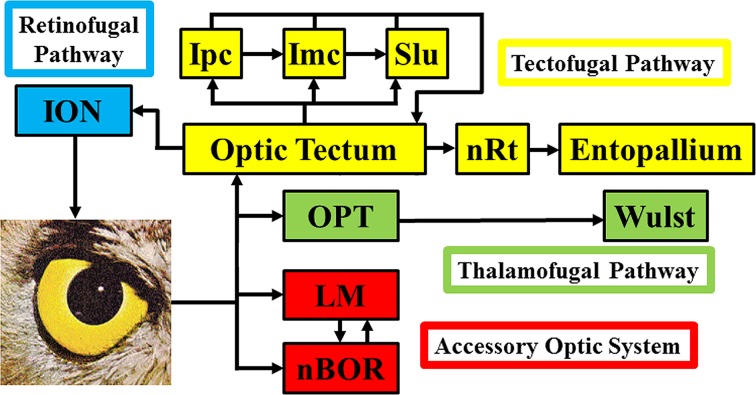
**Basic connections of the visual systems in birds**. ION, Isthmo-optic nucleus; Ipc/Imc, nucleus isthmi parvocellular/magnocellular; Slu, nucleus semilunaris; nRt, nucleus rotundus; OPT, principal optic nucleus of the thalamus; LM, nucleus lentiformis mesencephalic; nBOR, nucleus of the basal optic root.

Figure 1 shows a schematic of the visual connections in the avian visual system. The tectofugal pathway would be considered the major visual pathway as the optic tectum (TeO) receives more than 90% of retinal projections (Hunt and Webster, 1975; Remy and Güntürkün, 1991; Mpodozis et al., 1995). The TeO projects to the nucleus rotundus (nRt), which in turn projects to the entopallium (E) in the telencephalon (Benowitz and Karten, 1976; Nixdorf and Bischof, 1982; Miceli and Repérant, 1985; Karten and Shimizu, 1989; Bischof and Watanabe, 1997; Hellmann and Güntürkün, 1999; Laverghetta and Shimizu, 2003; Marín et al., 2003; Hellmann et al., 2004). Collectively, this pathway is involved in many visual behaviors and processes including brightness, color, pattern discrimination, and simple and complex motion (Frost and Nakayama, 1983; Remy and Güntürkün, 1991; Wang et al., 1993; Bischof and Watanabe, 1997; Luksch et al., 1998; Sun and Frost, 1998; Husband and Shimizu, 2001; Nguyen et al., 2004). The TeO is intimately connected with the isthmal nuclei, which includes the magnocellular and parvocellular parts of the nucleus isthmi (Imc and Ipc) and the nucleus semilunaris (SLu) (Hunt and Künzle, 1976; Brecha, 1978; Güntürkün and Remy, 1990; Hellmann and Güntürkün, 2001; Wang et al., 2004, 2006; Tömböl et al., 2006). These nuclei are involved in selective attention (Marín et al., 2003, 2007; Marin et al., 2012). The thalamofugal pathway is considered homologous to the geniculo-striate pathway in mammals and includes nuclei within the anterior dorsolateral thalamus collectively known as the principal optic nuclei of the thalamus (OPT), which projects to the visual Wulst (also known as the hyperpallium) (Karten et al., 1973; Karten and Shimizu, 1989; Shimizu and Karten, 1991; Medina and Reiner, 2000; Butler and Hodos, 2005; Reiner et al., 2005). The function of this pathway has been somewhat controversial (Martin, 2009), but it appears to play a role in spatial orientation (Michael et al., 2015), motion perception (Baron et al., 2007), and binocular vision (Pettigrew and Konishi, 1976). The nucleus of the basal optic root (nBOR) and the nucleus lentiformis mesencephalic (LM) are retinal-recipient nuclei (Karten et al., 1977; Reiner et al., 1979; Fite et al., 1981; Gamlin and Cohen, 1988; Wylie et al., 2014) collectively referred to as the Accessory Optic System (AOS) (Simpson, 1984), although technically the LM is a pretectal structure (Giolli et al., 2006). The AOS has a very specific function insofar as it is involved in the analysis of optic flow that results from self-motion and generating the optokinetic response (OKR) (Simpson, 1984; Simpson et al., 1988; Grasse and Cynader, 1990; Gamlin, 2006; Giolli et al., 2006). This is discussed in more detail below. Finally, in Figure 1 we also show the retinofugal pathway. The isthmo optic nucleus (ION), receives projections from the tectum and sends projections to the retina, thus creating a loop between retina, TeO and ION (Holden, 1968; Weidner et al., 1987; Wolf-Oberhollenzer, 1987). Numerous functions have been proposed for this pathway (for reviews see Repérant et al., 2006; Wilson and Lindstrom, 2011), which we tested through a detailed comparative analysis of ION size (Gutiérrez-Ibáñez et al., 2012).

## Hypertrophy of the LM in hummingbirds

**Figure 2:**
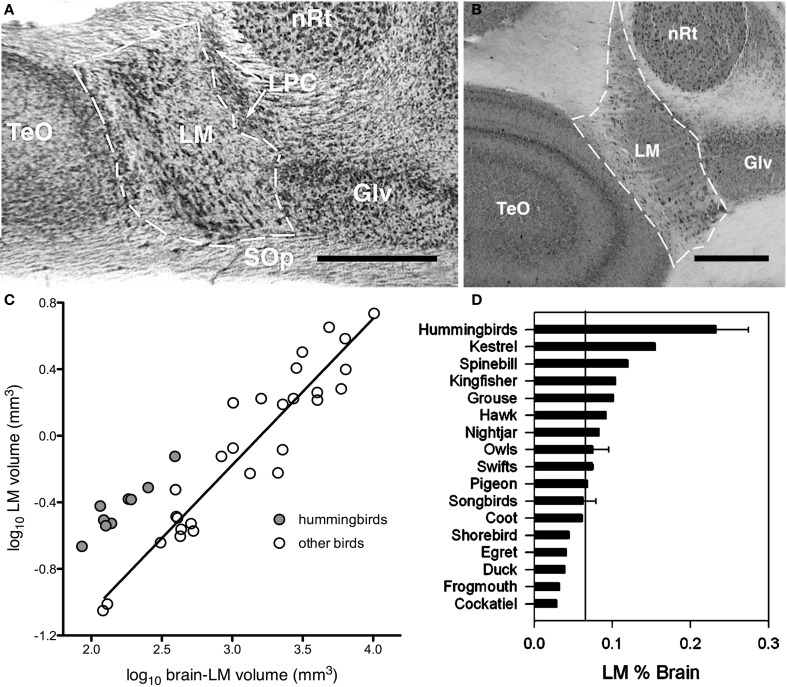
**Hypertrophy of the nucleus lentiformis mesencephalic (LM) in hummingbirds**. **(A,B)** Photomicrographs showing the location and borders of LM in coronal sections for a hummingbird (Fork-tailed woodnymph, *Thalurania furcate*) and a songbird (Eastern yellow robin, *Eopsaltria australis*). Although the brain of the songbird is much larger than that of the hummingbird, they share a similar LM volume. **(C)** Shows a scatter plot of the relative size of LM as a function of brain minus LM volume (log transformed). The hummingbirds are indicated by the gray circles and other birds by the white circles. The solid line indicates the least squares linear regression line for all species. **(D)** Bar graph of the relative size of LM expressed as a percentage of total brain volume. The solid line indicates the mean for all non-hummingbirds and the error bars indicate the standard deviations. TeO, optic tectum; LPC, nucleus laminaris precommissuralis; nRt, nucleus rotundus; Glv, lateral geniculate nucleus, ventral leaflet; SOp, stratum opticum. Scale bars = 0.5 mm (adapted from Iwaniuk and Wylie, 2007).

Assuming Jerison's Principle of Proper Mass, and given knowledge of the functions of specific visual pathways combined with knowledge of visual ecology and behavior, one can make predictions of the relative sizes of the visual nuclei in the brain. As mentioned above, the AOS is involved in the analysis of optic flow and the generation of the OKR to mediate retinal image stabilization. Iwaniuk and Wylie (2007) predicted that the nuclei of the AOS would be enlarged in hummingbirds to support their sustained hovering flight, which is unique among birds (Altshuler and Dudley, 2002). Hummingbirds beat their wings up to 50 times faster than other birds (Schuchmann, 1999), produce force during both up and down strokes rather than just up strokes (Warrick et al., 2005). Kinematically, the hovering flight of hummingbirds is unlike that of other birds, but is remarkably similar to that of some insects (Warrick et al., 2005). A critical feature of hovering is stabilization: hummingbirds are able to maintain a stable position in space, despite perturbations that must occur due to the inertia caused by wingbeats, and environmental factors such as wind gusts. Stabilization is controlled by several vestibular, visual, and proprioceptive reflexes, including the OKR (Wilson and Melvill Jones, 1979; for reviews see Ito, 1984; Melvill-Jones, 2000). To reiterate, the OKR is a visual following response to large moving visual stimuli (i.e., optic flow caused by self-motion) whereby eye, head, and body movements are made in the direction of motion to minimize the amount of visual motion across the retina. Lesions to either the nBOR or LM significantly impairs or outright abolishes the OKR (Fite et al., 1981; Gioanni et al., 1983a,b), and neurons in these nuclei have extremely large receptive fields and exhibit direction selectivity to optic flow stimuli (Burns and Wallman, 1981; Morgan and Frost, 1981; Gioanni et al., 1984; Winterson and Brauth, 1985; Frost et al., 1990). Most LM and nBOR neurons prefer extremely slow stimulus velocities on the order of about 1°/s (Burns and Wallman, 1981; Wylie and Crowder, 2000; Crowder et al., 2003) and as such are thought to provide the error signal that drives the OKR (Simpson, 1984; Simpson et al., 1988; Miles and Wallman, 1993). Given this, we hypothesized that both nBOR and LM would be hypertrophied in hummingbirds, compared with other birds, to meet the increased optic flow processing and OKR demands of hovering flight. We found that the LM, but not the nBOR, was significantly larger in hummingbirds compared to other birds (Figure 2). When expressed as a percentage of brain volume, the LM in hummingbirds was, on average, more than 3X larger than that of other birds (Figure 2D). Thus, we concluded that the OKR is critical for the unique ability of hummingbirds to hover, and this necessitated an increase in the size of the LM, as it is involved in mediating the OKR. This suggestion has recently been confirmed by Goller and Altshuler (2014). They filmed free-flight hummingbirds in a virtual reality environment to examine hovering in the presence of moving patterns. They found that hummingbirds lost positional stability and responded appropriately to the moving stimulus to minimize optic flow.

## Binocular vision and the wulst

**Figure 3:**
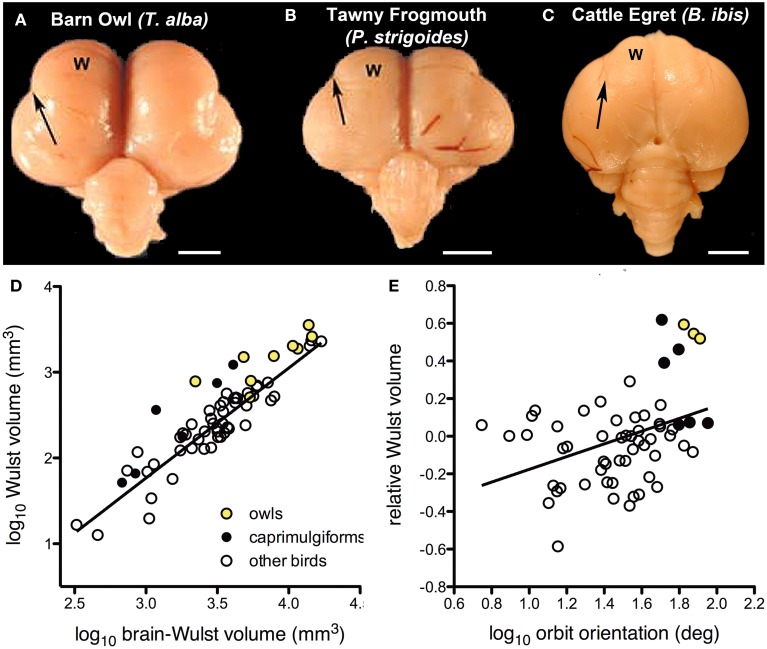
**Variation in the size of the visual Wulst (W) is related to binocular vision and stereopsis**. (**A**,**B** and **C**) respectively show dorsal views of the Barn Owl *(T. alba)*; Tawny Frogmouth *(P. strigoides)*; and the Cattle Egret *(B. ibis)*. The valecula, the lateral border of the Wulst, is indicated by the arrow. Scale bars = 5 mm. Adapted from Iwaniuk et al. (2006). **(D)** Shows a scatter plot Wulst volume as a function of brain minus Wulst volume. **(E)** Shows a scatterplot of Wulst volume relative to brain volume as a function of orbit orientation. The yellow circles indicate the owls (Strigiformes), black circles indicate Caprimuligiformes and the open circles are other species. The three species of Caprimulgiformes with the largest Wulst are the Oilbird *(S. caripensis)*, the Feline Owlet-nightjar *(A. insignis)*, and the Tawny Frogmouth *(P. strigoides)*. Adapted from Iwaniuk et al. (2008) with additional data from Gutiérrez-Ibáñez et al. (2013).

There is considerable variation in the size of the visual Wulst among birds and it appears have become enlarged to support global stereopsis associated with binocular vision (Iwaniuk and Hurd, 2005; Iwaniuk and Wylie, 2006; Iwaniuk et al., 2008). Based upon physiological and hodological evidence, the Wulst is considered the homolog of mammalian primary visual cortex (V1) (Karten et al., 1973; Pettigrew, 1979; Shimizu and Karten, 1993; Medina and Reiner, 2000; Husband and Shimizu, 2001; Reiner et al., 2005). Based on external morphology of the brain, owls appear to have a greatly hypertrophied Wulst compared to other groups of birds (Figures 3A,C). In owls, this coincides with a large frontal binocular overlap on the order of 50° (Martin, 1984; Pettigrew and Konishi, 1984; Wylie et al., 1994), which is much greater than that measured in other birds (Katzir and Martin, 1999; Martin and Coetzee, 2004). Electrophysiological studies in owls show that, as in V1, the Wulst is retinotopically organized and neurons are tuned to spatial frequency and orientation. Furthermore, the majority of cells in the Wulst have receptive fields located in the area of binocular overlap. Most cells (about 85%) are binocular, and sensitive to retinal disparity (Pettigrew and Konishi, 1976; Pettigrew, 1978, 1979; Porciatti et al., 1990; Wagner and Frost, 1993; Nieder and Wagner, 2000, 2001). Binocular neurons are present in the Wulst of other species, but they are not as numerous as they are in owls (Pettigrew, 1978; Wilson, 1980; Denton, 1981; Michael et al., 2015). Together, this suggests that one of the primary functions of the visual Wulst is to mediate binocular vision and/or stereopsis. In support of this hypothesis, Iwaniuk and Wylie (2006) showed that an enlarged visual Wulst seems to have evolved in concert with binocular vision in other nocturnal birds as well. Both the Owlet-Nightjars (genus *Aegotheles*) and frogmouths (genus *Podargus*) are thought to possess stereopsis (Pettigrew, 1986) and have large areas of binocular overlap rivaling that of the owls (Pettigrew and Konishi, 1984; Wallman and Pettigrew, 1985; Martin et al., 2004a). The Wulst is also quite large in these birds, showing a similar degree of hypertrophy as seen in owls (Figures 3A,B,D) (Iwaniuk and Wylie, 2006; Iwaniuk et al., 2008), including a prominent pattern of lamination. The closely related nightjars and potoos (genus *Nyctibius*) do not share this Wulst hypertrophy and have a much narrower binocular visual field (Martin et al., 2004a,b).

The relationship between the size of the Wulst and degree of binocular vision seems to hold beyond these birds with a large degree of binocular overlap. Using a data set including 58 different species, Iwaniuk et al. (2008) examined the relationship between the size of the Wulst and binocular vision using orbit orientation as a proxy for binocular overlap (Figure 3E). The relative size of the Wulst was significantly correlated with orbit orientation (Figure 3E), but relative TeO size was not. Although these multiple lines of evidence indicate that the Wulst is enlarged in species to support binocular vision and global stereopsis, there are some clear exceptions. The oilbird (*Steatornis caripensis*) has a large binocular overlap (Pettigrew and Konishi, 1984) and a hypertrophied Wulst (Figure 3D), however, an electrophysiological study failed to find any binocular neurons in the Wulst (Pettigrew and Konishi, 1984). Iwaniuk and Wylie (2006) suggested that binocular vision has been lost in the Oilbird as a consequence of roosting deep within caves and the moderately enlarged Wulst could therefore be a “carryover” from a stereoscopic ancestor. To further complicate this link between relative Wulst size and binocularity, hawks, eagles, and falcons have an abundance of binocular disparity sensitive neurons in the Wulst (Pettigrew, 1978) and stereopsis (Fox et al., 1977), but have a narrow binocular field (Wallman and Pettigrew, 1985; Katzir and Martin, 1999) and a relatively small Wulst (Iwaniuk et al., 2008). Some authors have even suggested that the Wulst has different functions in frontally vs. laterally eyed birds (Michael et al., 2015). Last, it also worth noting that the Wulst is not an exclusively visual structure; the rostral Wulst receives somatosensory projections (Funke, 1989; Wild, 1997; Medina and Reiner, 2000; Manger et al., 2002). In species that forage using tactile information originating in the beak, the rostral Wulst is hypertrophied (Pettigrew and Frost, 1985). One possible explanation for the enlargement of the oilbird's Wulst could therefore be a reflection of increased reliance on somatosensory information from its rictal bristles. This caveat in itself suggests one should be cautious with the general approach to using Jerison's Principle of Proper Mass given that many neural structures can be heterogeneous.

## Variation in the size of the isthmo-optic nucleus (ION)

In most studies using Jerison's Principle of Proper Mass, including our studies of the LM (Iwaniuk and Wylie, 2007) and Wulst (Iwaniuk and Wylie, 2006; Iwaniuk et al., 2008) outlined above, the correlation between a structure and a behavior is established with an *a priori* knowledge that the structure is related to the generation of the behavior or sensory modality. Gutiérrez-Ibáñez et al. (2012) examined variation in the size of the ION applying the opposite strategy: the relative size of the structure was used to determine the function of the ION. There have been numerous studies of the ION in birds with little consensus on its function (for reviews see Repérant et al., 2006; Wilson and Lindstrom, 2011). The various functions proposed for the ION include: shifting of visual attention (Rogers and Miles, 1972; Catsicas et al., 1987; Uchiyama, 1989; Ward et al., 1991; Clarke et al., 1996; Uchiyama et al., 1998), saccadic suppression (Holden, 1968; Nickla et al., 1994) enhancement of peripheral vision (Marin et al., 1990), modulation of temporal processing (Knipling, 1978), feeding/pecking (Shortess and Klose, 1977; Weidner et al., 1987; Repérant et al., 1989; Hahmann and Güntürkün, 1992), and detection of aerial predators (Wilson and Lindstrom, 2011).

**Figure 4:**
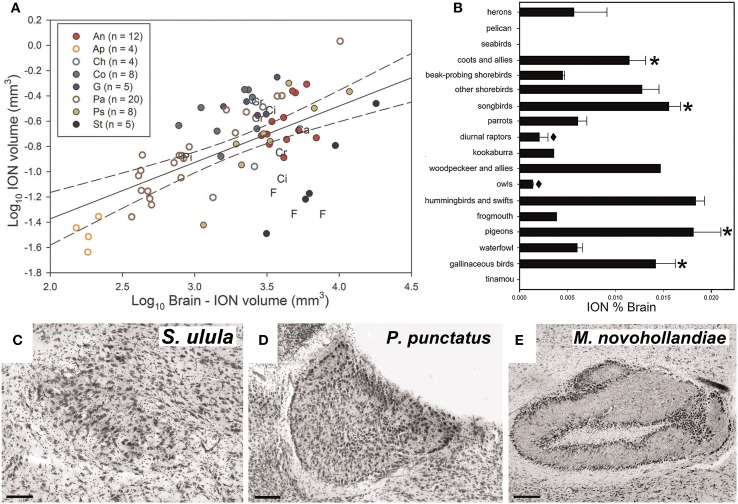
**Variation in the volume and complexity of the isthmo optic nucleus (ION)**. **(A)** Shows a scatterplot of ION volume plotted as a function of brain minus ION volume (log transformed). *n* indicates to the number of species measured in each order. An, Anseriformes (red full circles); Ap, Apodiformes (empty orange circle); Ca, Caprimulgiforms; Ch, Charadriiforms (empty light blue circle); Ci, Ciconiiformes; Co, Columbiforms (dark green full circles); Cr, Coraciiforms; F, Falconiforms; G, Galliformes (dark blue full circle); Gr, Gruiformes; Pa, Passerifomes (empty brown circles); Pi, Piciforms; Ps, Psittaciformes (full yellow circle); St, Strigiforms (full black circle). **(B)** Shows a bar graph of the relative size of ION expressed as a percentage of total brain volume for the different groups of birds. The error bars indicate standard error. The asterisk (^*^) indicates the groups in which a lower field myopia has been described (Martin, 1986, 1993; Hodos and Erichsen, 1990; Schaeffel et al., 1994). The black diamond (♦) indicates species where a lack of lower field myopia has been described (Murphy et al., 1995). **(C–E)** Show variation in the complexity of the ION. The ION complexity representative of categories 1, 3, and 5 (most complex) are, respectively, shown in **(C)** (Northern Hawk-Owl, *S. Ulula*), **(D)** (Spotted Pardalote, *P. punctatus*), and **(E)** (Superb Lyrebird, *M. novaehollandiae*). Scale bars, 100 μm in **(C,D)**, 200 μm in **(E)** (Adapted from Gutiérrez-Ibáñez et al., 2012).

Gutiérrez-Ibáñez et al. (2012) examined interspecific variation in the relative size of ION in an attempt to address its function. For example, if the ION is an essential component of pecking behavior, then we predicted that species that feed on the ground, such as granivorous finches and galliforms, would have an enlarged ION. Alternatively, if the ION is critical for the detection of aerial predators, then prey species should have larger ION volumes than predatory species. Across 81 species, there was considerable variation in the relative size of the ION (Figure 4A). In some birds, including basal, paleognathous species, the ION was not apparent in Nissl stained sections When expressed as a percentage of total brain volume, the ION was quite small in owls and diurnal raptors, but quite large in coots, some shorebirds, songbirds, hummingbirds, woodpeckers, pigeons, and galliforms (Figure 4B).

The ION varied not only in size but also the complexity of its visible morphology. The complexity was assigned to one of five categories representing and increasing degree of complexity. For example in category 1, the ION is an evenly distributed mass of cells with somewhat indistinct borders (Figure 4C). In category 3, the ION is characterized by a sharper border with a distinct layer of cells that encapsulates the rest of the nucleus (Figure 4D). In category 5, all cells appear to be organized into distinct layers with a clearly recognizable neuropil between the layers (Figure 4E). Generally speaking, the complexity of the ION was correlated with size, such that birds with a relatively large ION also had a more complex ION. This emphasizes that a strict interpretation of the Principle of Proper Mass (i.e., considering only size) may miss other neuronal features that may also be indicative of a processing capacity.

Based on these data, Gutiérrez-Ibáñez et al. (2012) proposed an alternative theory for ION function. Many of the birds that have a relatively large ION (and relatively complex ION; see below) also have a lower field myopia including: pigeons (Fitzke et al., 1985), songbirds (Martin, 1986), galliforms (Schaeffel et al., 1994), and gruiforms (Hodos and Erichsen, 1990), all which have relatively large IONs (Figure 4B). In contrast, owls and diurnal raptors, both of which have small IONs (Figure 4B), do not have a lower field myopia (Murphy et al., 1995). (Gutiérrez-Ibáñez et al., 2012) therefore suggested that the ION is involved in switching attention from an emmetropic to a myopic part of the retina (i.e., switching from long range to close range). Gutiérrez-Ibáñez et al. (2012) further linked this to feeding behavior. Birds with large IONs (chickens, pigeons, songbirds, woodpeckers, hummingbirds) feed close to the substrate, which can include the ground, flowers and tree trunks. Many of these birds have a lower field myopia, thus the substrate from which they are feeding would be fall in the myopic part of the retina. In contrast, the birds with smaller IONs feed far from the substrate, or have non-visually guided foraging behaviors (e.g., somatosensory based). Owls and diurnal raptors feed by perch hunting or feeding on the wing (Jaksić and Carothers, 1985) and are therefore some distance from the substrate. The reduced size of the ION in herons and the apparent absence of an ION in seabirds and a pelican (Figure 4B) also fits this hypothesis, as seabirds and pelicans usually dive into the water to catch fish, while herons have longs legs that keep their eyes at a considerable distance from the ground when foraging (Martin and Katzir, 1994).

## Lack of hypertrophy in the tectofugal pathway

**Figure 5:**
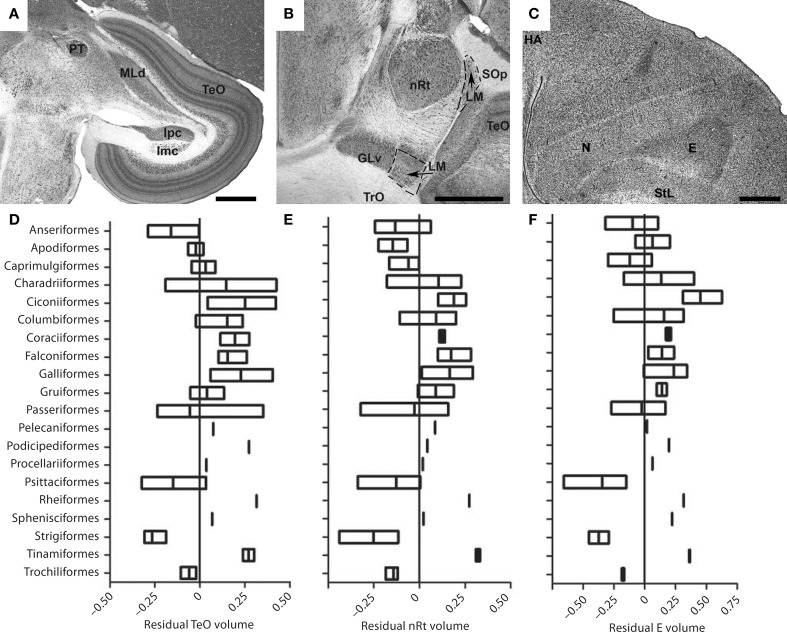
**Variation in the size of structures in the tectofugal pathway**. **(A–C)** Show Nissl stained sections highlighting the major nuclei of the tectofugal pathway: the optic tectum (TeO) **(A)**, the nucleus rotundus (nRt) **(B)** and the Entopallium (E) **(C)**. The sections in **(A,B)** are from an Eastern Yellow Robin *(E. australis)* whereas that in **(C)** is from a Short-billed Dowitcher *(L. griseus)*. GLv, ventral leaflet of the lateral geniculate nucleus; GP, globus pallidus; HA, hyperpallium apicale; Imc, nucleus isthmi magnocellularis; Ipc, nucleus isthmi parvocellularis; LM, nucleus lentiformis mesencephali; MLd, nucleus mesencephalicus lateralis, pars dorsalis; N, nidopallium; PT, nucleus pretectalis; SOp, stratum opticum; StL, lateral striatum; TrO, optic tract. **(D–F)** Show boxplots showing the variation of the relative size of TeO **(D)**, nRT **(E)**, and Entopallium **(F)**. Scale bars = 1 mm (Adapted from Iwaniuk et al., 2010).

Despite the fact that the tectofugal pathway (TeO, nRt, E; see Figures 5A–C) is regarded as the “main” visual pathway and is the primary source of visual input to the avian brain, there is relatively little variation in the relative size of the pathway as a whole or each of the brain regions that comprise this pathway (Iwaniuk et al., 2010). All three structures, TeO, nRt, and E, were somewhat smaller in owls, parrots, and waterfowl (Figures 5D–F). Although not included in Iwaniuk et al. (2010), Martin et al. (2007) found that the kiwi (*Apteryx mantelli*) has an even smaller TeO and likely represents a case of tectofugal hypotrophy. This may not reflect a reduction in the tectofugal regions *per se*, but rather an expansion of other regions and pathways. Waterfowl, parrots and owls all have an enlarged telencephalon (Portmann, 1947; Iwaniuk and Hurd, 2005), but have enlarged regions within the telencephalon other than the E. The apparently small tectofugal pathway may thus be a result of an enlarged telencephalon in these groups. At the other end of the spectrum, no species appeared to have a hypertrophied tectofugal pathway.

The isthmal nuclei (Imc, Ipc, Slu), which are closely associated with the tectofugal pathway, scaled with negative allometry relative to brain size, but had isometric (i.e., 1:1) relationships with TeO and nRt (Gutiérrez-Ibáñez et al., 2014). Thus, it seems that all the intimately connected nuclei within the tectofugal system have evolved in concert and that variation in the size of any one is generally accompanied by a similar degree of variation in the others.

The lack of hypertrophy in the tectofugal pathway is in marked contrast to what we observed in LM, Wulst and ION. The lack of such hypertrophy could reflect the heterogeneous organization of the tectofugal pathway, insofar as color, motion, and form are all processed in this pathway (Frost et al., 1988; Wang et al., 1993; Bischof and Watanabe, 1997; Sun and Frost, 1998; Nguyen et al., 2004; Xiao et al., 2006; Xiao and Frost, 2009). The cells within the tectofugal regions are tuned to specific types of visual functions. Within nRt, for example, neurons are tuned to 3D motion (“looming”), 2D motion, luminance and color, with each of these components represented in a separate subregion of the nucleus (Wang et al., 1993). Similarly, form and visual motion are, respectively, represented in rostral and caudal margins of E (Nguyen et al., 2004). These subdivisions cannot be discerned in Nissl stained brain sections, but species could vary in the proportional size of these motion, form, and color-regions, depending on their ecology and behavior. Thus, some birds could require more cells responsive to motion processing vs. color. The relative sizes within nRt and E that respond to motion could then be enlarged at the expense of the color regions without having an effect on the overall size. Neurochemical markers that delineate these subregions or neurophysiological data for a broader range of species would enable us to test this hypothesis in the future.

## Brain–behavior relationships in the avian auditory system

**Figure 6:**
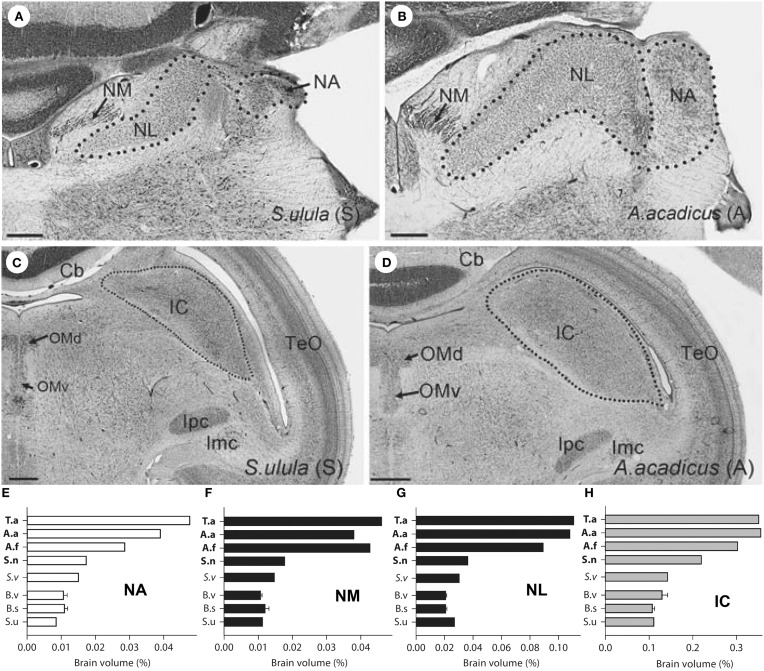
**(A–D)** Show photomicrographs of coronal section of auditory structures for a symmetrically-eared owl (Northern Hawk Owl, *S. ulula*) **(A,C)** and an asymmetrically-eared owl (Northern Saw-Whet Owl, *A. acadicus*) **(B–D)**. **(A,B)** Emphasize the size differences for the nucleus laminaris, angularis, and magnocellularis (NL, NA, NM) whereas **(C,D)** depict the size difference with respect to the inferior colliculus (IC). TeO, Optic tectum; Ipc, parvocellular part of the nucleus isthmi; Imc, magnocellular part of the nucleus isthmi; Cb, cerebellum; OMd/v, dorsal/ventral parts of the oculomotor nucleus. **(E–H)** Are bar graphs showing the sizes of NA **(E)**, NM **(F)**, NL **(G)**, and IC **(H)** expressed as a percentage of total brain volume for eight species of owls. Species abbreviations T.a, Barn owl (*T. alba*); A.a, Northern Saw-Whet owl (*A. acadicus*); A.f, Short-Eared Owl (*A. flammeus*); S.n, Great Gray Owl (*S. nebulosa*); S.v, Barred Owl (*S. varia*); B.v, Great Horned Owl (*B. virginianus*); B.s, Snowy Owl (*B. scandiacus*); S.u, Northern Hawk owl (*S.ulula*). Each species was classified as having a high degree of vertical ear asymmetry (T.a, A.a, A.f, S.n), a moderate degree of ear asymmetry (S.v) or symmetrical ears. (B.v, B.s, S.u) (Adapted from Gutiérrez-Ibáñez et al., 2011).

Investigations of brain–behavior relationships in birds is not restricted to visual systems. The auditory system has also been examined, especially in owls because of their remarkable sound localization ability, unique morphological specializations, and rather sophisticated, adaptive neural circuitry (Schwartzkopff and Winter, 1960; Payne, 1971; Knudsen et al., 1979; Knudsen, 1999; Takahashi et al., 2003; Whitchurch and Takahashi, 2006; Takahashi, 2010). A rather unique feature that sets some owls apart from others with respect to sound localization is the presence of vertically asymmetrical ears, which has evolved independently several times in owls (Norberg, 1977, 2002). This vertical ear asymmetry is particularly important for localizing sounds in elevation. To localize sound, neurons within the external nucleus of the inferior colliculus (ICx) of the midbrain are tuned to auditory space, but these neurons vary in their receptive fields between asymmetrically and symmetrically eared owls. In owls with vertically asymmetrical ears, these neurons have receptive fields that are restricted in both elevation and azimuth, whereas in owls with vertically symmetrical ears, they are restricted only in azimuth (Knudsen et al., 1977; Knudsen and Konishi, 1978a,b; Wise et al., 1988; Volman and Konishi, 1990). The tuning of both elevation and azimuth enables asymmetrically eared owls to accurately capture prey in complete darkness based solely on acoustic cues whereas symmetrically eared owls cannot (Payne, 1971). In barn owls, the azimuthal and elevational components of a sound locale are computed using interaural time differences (ITDs) and interaural level differences (ILDs), respectively (Knudsen and Konishi, 1979, 1980; Moiseff and Konishi, 1981; Moiseff, 1989). Furthermore, ITDs and ILDs are processed in two separate pathways from the cochlear nuclei to the ICx (Moiseff and Konishi, 1983; Takahashi et al., 1984; Takahashi and Konishi, 1988a,b; Adolphs, 1993; Mazer, 1998). The cochlear nerve projects directly to two nuclei in the brainstem: nucleus angularis (NA) and nucleus magnocellularis (NM) (Carr and Boudreau, 1991). Processing of ILD begins in NA, whereas ITD processing begins with NM (Figures 6A,B). NM projects bilaterally to nucleus laminaris (NL) where ITD is first calculated. The ITD and ILD pathways eventually project to different parts of the inferior colliculus (IC) (Figures 6C,D) and converge in ICx (Knudsen and Knudsen, 1983; Takahashi et al., 1984; Carr and Konishi, 1990). Given that owls with asymmetrical ears exploit ILDs to compute the elevation of a sound source, Gutiérrez-Ibáñez et al. (2011) hypothesized that the structures in involved in computing ILDs, including NA and the IC, should be larger in owls with vertical asymmetrical ears, whereas there should be no differences in the structures that process only ITD (NM, NL). However, all nuclei in the ITD and ILD pathways were larger in the owls with a vertical ear asymmetry (Figure 6). This increase in size of nuclei in both ILD and ITD pathways might be related to a general expansion of hearing range in asymmetrically eared owls. In symmetrically eared owls, audibility deteriorates rapidly above 6 kHz whereas in asymmetrically eared owls the high-frequency cutoff lies between 10 and 13 kHz (Konishi, 1973; Van Dijk, 1973; Dyson et al., 1998). These higher frequency are effectively shadowed by the head such that ILD varies with elevation (Norberg, 1978; Volman and Konishi, 1990). That is, in order to use ILDs to detect localize sound, an asymmetrically eared owl must have high sensitivity to high frequencies. Thus, the expansion of the audible range would explain not only the equal enlargement of the ILD pathway, but also the hypertrophy of all auditory nuclei and this has happened several times throughout the evolutionary history of owls. Based on these anatomical differences in owls, one would predict that harriers (*Circus* sp.) also have enlarged auditory nuclei. Harriers are diurnal raptors that have an owl-like facial ruff, hunts in a similar fashion to short-eared owls (*Asio flammeus*) and are capable of resolving azimuth at a similar acuity to owls (Rice, 1982), but neuroanatomical studies of any harrier species are wanting.

## Hypertrophy in the somatosensory system

**Figure 7:**
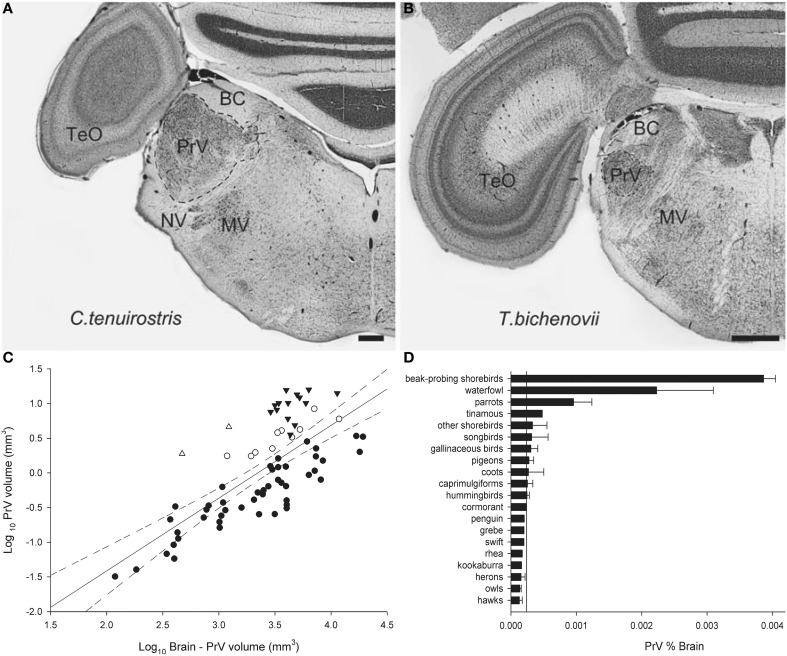
**Photomicrographs of coronal sections through the principal sensory nucleus of the trigeminal nerve (PrV) of a somatosensory specialist (A, Long-Billed Corella, *C. tenuirostris*) and a non-specialist (B, Double-Barred Finch, *T. bichenovii*)**. TeO, optic tectum; BC, brachium conjunctivum; NV, root of the trigeminal nerve; MV, motor nucleus of the trigeminal nerve. **(C)** Shows a scatterplot of PrV volume as a function of brain minus PrV volume for all species examined. Waterfowl are indicated by black triangles, beak-probing shorebirds by white triangles, parrots by white circles, and non-specialists by black circles. **(D)** Is a histogram of the relative size of PrV expressed as a percentage of total brain volume. The solid line indicates the mean for all non-specialists and the error bars indicate standard deviations. Scale bars = 600 μm (Adapted from Gutiérrez-Ibáñez et al., 2009).

Finally, we will illustrate an example of Jerison's Principle of Proper Mass as applied to the somatosensory system. Beak size and shape varies immensely among bird species in relation to their foraging behavior and diet. In addition to beak shape, the number, type and distribution of mechanoreceptors also varies among species (Gottschaldt, 1985) and these features reflect feeding behavior. For example, in beak-probing shorebirds mechanoreceptors are numerous and concentrated in the tip of the beak (Bolze, 1968; Pettigrew and Frost, 1985) whereas in ducks and geese they are more widely distributed across the beak, as well as on the tongue (Berkhoudt, 1979). The beak mechanoreceptors are innervated by the trigeminal nerve (nV; Dubbeldam and Karten, 1978) of which one of the main targets is the principal sensory nucleus of the trigeminal nerve (PrV) (Figure 7) (Zeigler and Witkovsky, 1968; Silver and Witkovsky, 1973; Kishida et al., 1985; Dubbeldam, 1998). PrV also receives projections from the facial (nVII), glossopharyngeal (nIX) and hypoglossal (nXII) nerves and thus the PrV gathers information from the beak, palate, tongue, and pharynx (Dubbeldam et al., 1979; Wild, 1981; Bout and Dubbeldam, 1985; Woodson et al., 1995). PrV is hypertrophied in several taxa: beak-probing shorebirds, waterfowl, parrots, and kiwi (Stingelin, 1965; Boire, 1990; Dubbeldam, 1998; Gutiérrez-Ibáñez et al., 2009; Cunningham et al., 2013) (Figures 7C,D). Thus, the enlargement of the PrV had evolved at least three times in birds to support three types of feeding behavior, beak-probing (shorebirds and kiwi), filtering (waterfowl), and seed husking (parrots), which all demand processing of mechanoreceptor information from the beak (Zweers et al., 1977, 1994; Berkhoudt, 1979; Gerritsen and Meiboom, 1985; Gottschaldt, 1985; Zweers and Gerritsen, 1996; Piersma et al., 1998; Cunningham et al., 2013).

PrV projects to the somatotopically organized nucleus basorostralis (Bas) in the telencephalon (Witkovsky et al., 1973; Berkhoudt et al., 1981; Dubbeldam et al., 1981; Wild et al., 1985; Wild and Farabaugh, 1996). The size of Bas varies with that of PrV, but species with an enlarged PrV do not necessarily have an enlarged Bas (Cunningham et al., 2013). Waterfowl, kiwi, and beak-probing shorebirds all have an enlarged PrV and Bas, but parrots only appear to have an enlarged PrV. As with some of the aforementioned comparisons of telencephalic brain regions, this could reflect the expansion of other telencephalic regions in parrots, such as the nidopallium and mesopallium (Iwaniuk and Hurd, 2005), or the fact that Bas is receiving other forms of sensory input. Nevertheless, the Principle of Proper Mass certainly applies to the somatosensory system in birds.

## Trade-offs

If you are a somatosensory or auditory specialist, does this come at the expense of sacrificing another sensory system? Brain tissue is among the more energetically expensive as it requires almost an order of magnitude more energy per unit weight than many other tissues (Mink et al., 1981) and is not only expensive to use, but also to maintain (Niven and Laughlin, 2008). The large energy requirements of the brain has been proposed to be a major factor in the evolution of brains in vertebrates (Aiello and Wheeler, 1995; Striedter, 2005; Fonseca-Azevedo and Herculano-Houzel, 2012). The expensive brain hypothesis predicts that relatively large brains can evolve only when either energy input increases (Aiello and Wheeler, 1995; Isler and van Schaik, 2006a) or there is a trade-off that implies reduction of another expensive tissue such as the digestive tract in primates (Aiello and Wheeler, 1995) or the pectoral muscle in birds (Isler and van Schaik, 2006b). Recent selection experiments in fish seem to confirm this hypothesis as selection for larger brains results in the reduction of gut size in only a few generations (Kotrschal et al., 2013). Concordantly, it has also be proposed that trade-offs occur within the brain so that expansion of one area is accompanied by reduction in another. So far, evidence for this trade-off in neural tissue comes mostly from sensory systems. For example, fish species that live permanently in caves have reduced visual system and an expanded lateral line system when compared with surface-dwelling species (Poulson and White, 1969; Niven and Laughlin, 2008; Soares and Niemiller, 2013). In mammals, Baron et al. (1996) found that there is a tradeoff between the relative sizes of auditory and visual structures in the mesencephalon in bats (see also Iwaniuk et al., 2006), and Eisenberg (1981) suggested that a similar trade-off between visual and auditory pathways may occur in tenrecs, which use echolocation and have small eyes. Further, some subterranean mammals, like the star-nosed mole (*Condylura cristata*) or the blind mole rats (*Spalax ehrenbergi*), have reduced thalamo-cortical visual systems and an expanded somatosensory representation, particularly of the trigeminal system (Cooper et al., 1993; Catania and Kaas, 1995).

**Figure 8:**
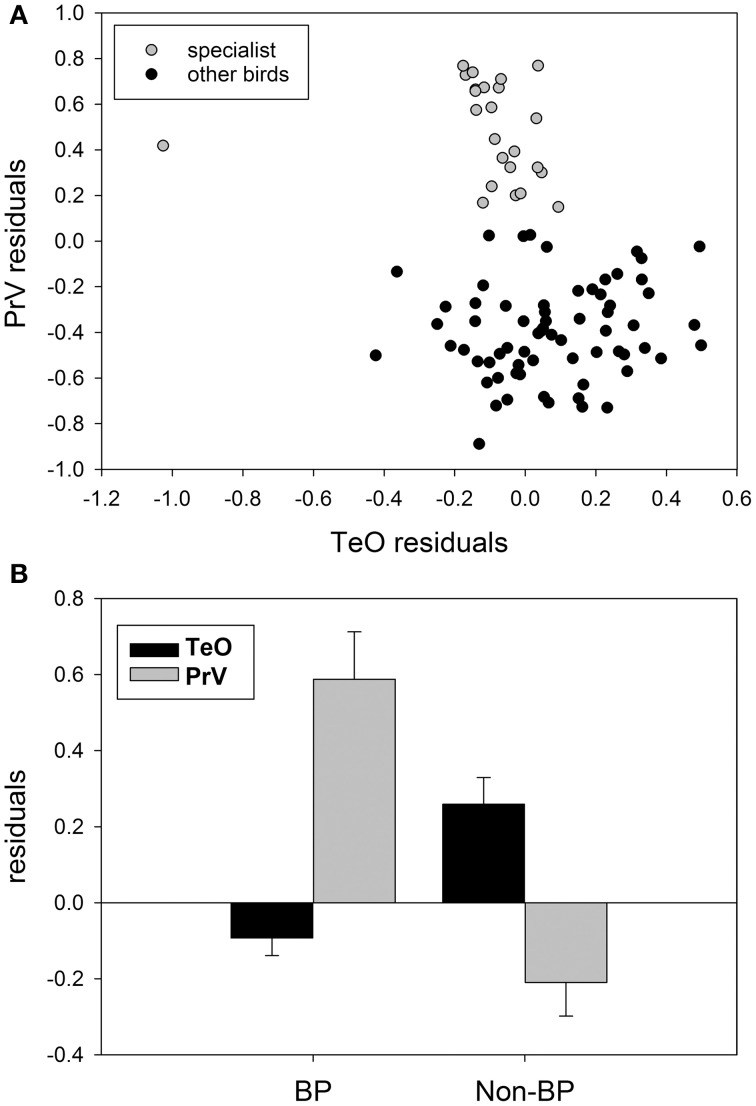
**(A)** Shows the size of the principal sensory nucleus of the trigeminal nerve (PrV) as a function of the optic tectum (TeO) for somatosensory specialists: parrots waterfowl, beak-probing shorebirds and the kiwi (gray circles) and other birds (black circles). **(B)** Shows a comparison of the relative size of the TeO and PrV for beak-probing (PB) shorebirds and non-beaking-probing (Non-BP) shorebirds. Data from Iwaniuk et al. (2010), Gutiérrez-Ibáñez et al. (2009), and Cunningham et al. (2013).

Although there has been no clear demonstration of trade-offs between sensory systems in birds, there is some evidence that this phenomenon applies to avian sensory systems as well. For example, several groups present a tendency similar to subterranean mammals mentioned above, with a trade-off between the size of visual and trigeminal/somatosensory systems. First, as discussed above, waterfowl, parrots, and kiwi all have an enlarged trigeminal system and a small tectofugal pathway (Figure 8A) (Martin et al., 2007; Iwaniuk et al., 2010; Cunningham et al., 2013; Gutiérrez-Ibáñez et al., 2014). An extreme case of this trade-off within waterfowl could be the extinct species *Talpanas lippa* (Iwaniuk et al., 2009), which has a greatly reduced optic foramen and an extremely enlarged maxillo-mandibular (nV) foramen, much larger than any other waterfowl or bird. Second, within the order Charadriformes, there is a clear separation of species into those with a large trigeminal and a small tectofugal pathway and those with a large tectofugal and a small trigeminal pathway (Figure 8B). This separation reflects whether they are beak probing species or not. The beak probing sandpipers have a greatly expanded trigeminal system and a small TeO compared to the non-beak probing species (e.g., plovers, terns), which have a much smaller PrV and a larger TeO. One could even argue that owls and a subset of caprimulgiforms are yet another example of a trade-off, but within a single sensory domain: vision. Owls, frogmouths, and owlet-nightjars have a greatly enlarged thalamofugal system, with a correspondingly smaller tectofugal system (Iwaniuk and Wylie, 2006; Iwaniuk et al., 2010; Gutiérrez-Ibáñez et al., 2013).

Taken together this data suggest that in birds, like in other vertebrates, there are constraints in the evolution of sensory systems such that the enlargement of one sensory pathway is accompanied by the diminution of another sensory pathway. More detailed analyses of a wider range of species is needed to address these contingencies and to determine when and how rapidly these changes occur in evolutionary time. It is worth noting that although sensory trade-offs play a significant role in the evolution of sensory systems, it is certainly not the only factor any more so than phylogeny, allometry or behavior. In the case of the visual system of owls for example, the hypotrophy of the tectofugal pathway is probably related to a reduction in the number of retinal ganglion cells, which, in turn, is likely a result of the nocturnal history of the clade (Gutiérrez-Ibáñez et al., 2013). Thus, sensory trade-offs can only be understood in an integrative context that combines the functional organization of the sensory pathways with anatomy, behavior and phylogeny.

## Conclusion

An emerging pattern from the studies reviewed here is that changes in the size and cytoarchitecture of different neural structures occur repeatedly and these changes are largely independent of phylogeny. This is true for almost all the examples reviewed including: PrV (Gutiérrez-Ibáñez et al., 2009; Cunningham et al., 2013), visual wulst (Iwaniuk and Wylie, 2006; Iwaniuk et al., 2008), and the auditory system in asymmetrically eared owls (Gutiérrez-Ibáñez et al., 2011). The majority of these differences reflect “grade shifts” among clades of birds and likely occurred fairly early in the diversification of modern birds. For example, the expansion of PrV in waterfowl likely occurred at or close to the divergence between Galliformes and Anseriformes, which is estimated to be 65 million years ago (Jarvis et al., 2014). With recent advancements in avian genomics of birds (Jarvis et al., 2014; Koepfli et al., 2015), it is now possible to test the relationship between genes and neuroanatomy to obtain insight into the underlying molecular mechanisms responsible for species variation in brain anatomy. Recently, Schneider et al. (2014) showed that *Piezo2* is upregulated in waterfowl compared with galliforms and that this upregulation is related to increases in the number of large diameter fibers in the trigeminal nerve, expansion of PrV and increases tactile sensitivity. If *Piezo2* is an essential component of regulating tactile sensitivity, then it might also be upregulated in parrots, beak-probing shorebirds and kiwi. Similarly, the evolution of a vocal control system is associated with differential expression of two genes involved in axonal guidance (Wang et al., 2015) and even the evolution of novel genes in songbirds (Wirthlin et al., 2014). These two recent examples highlight the strengths and importance of incorporating gene regulation into comparative neuroanatomy to address not only what species differences are present, but also how they have occurred. Now that we are gaining a much more in depth understanding of anatomical variation in the avian brain, we can apply bioinformatics approaches (Mello and Clayton, 2015) to address mechanistic questions, such as “How and why do owls have such an enlarged hyperpallium?.” By integrating molecular mechanisms with evolutionary patterns, we will achieve a far deeper understanding of the evolution of the avian brain and behavior.

### Conflict of interest statement

The editor Jorge Mpodozis declares that, despite having collaborated on an article with authors Cristian Gutiérrez-Ibáñez and Andrew Iwaniuk in 2013, the review was conducted objectively. The authors declare that the research was conducted in the absence of any commercial or financial relationships that could be construed as a potential conflict of interest.
